# ^123^I-ioflupane brain SPECT and ^123^I-MIBG cardiac planar scintigraphy combined use in uncertain parkinsonian disorders

**DOI:** 10.1097/MD.0000000000006967

**Published:** 2017-05-26

**Authors:** Susanna Nuvoli, Angela Spanu, Maria Rita Piras, Antonio Nieddu, Aldo Mulas, Gaia Rocchitta, Grazia Galleri, Pier Andrea Serra, Giuseppe Madeddu

**Affiliations:** a Nuclear Medicine Unit; b Neurology Unit, Department of Clinical and Experimental Medicine, University of Sassari, Sassari, Italy; c Geriatric Unit, Policlinico Sassarese; d Pharmacology Unit; e Internal Medicine Unit, Department of Clinical and Experimental Medicine, University of Sassari, Sassari, Italy.

**Keywords:** ^123^I-ioflupane SPECT, ^123^I-MIBG cardiac scintigraphy, uncertain parkinsonism

## Abstract

We evaluated the clinical usefulness of the combined use of ^123^I-ioflupane brain single photon emission computed tomography (SPECT) and ^123^I-metaiodobenzylguanidine (MIBG) cardiac scintigraphy in discriminating uncertain parkinsonism with vascular lesions in striatal nuclei at magnetic resonance imaging (MRI). Forty-three consecutive patients with uncertain parkinsonism and vascular lesions at MRI in striatal nuclei were retrospectively evaluated; the uncertain differential diagnosis was between Parkinson's disease and vascular parkinsonism (PD/VP) in 22 patients, between PD and other neurodegenerative parkinsonism (PD/PS) in 11 patients and between Lewy body dementia and Alzheimer disease (LBD/AD) in the remaining 10 cases. All patients underwent ^123^I-ioflupane SPECT with striatal dopaminergic activity determination as binding potentials (BP; cut-off: 3.3). ^123^I-MIBG cardiac planar scintigraphy was performed 2 weeks later, in early (15 minutes) and delayed (240 minutes) phases also calculating heart to mediastinum (H/M) ratio (cut-off: 1.56). ^123^I-Ioflupane uptake was normal in 9 patients with BP values >3.3, while it was reduced in 34/43 cases with BP values <3.3 at least in one of the striatal nuclei. ^123^I-MIBG uptake was normal in 21/43 patients (5 of whom with normal and 16 with ^123^I-ioflupane striatal defects) showing the H/M ratio >1.56 in all cases; the uptake was reduced in 22/43 cases, (4 of whom were normal and 18 were with ^123^I-ioflupane striatal defects) with the H/M ratio <1.56 in all cases. No statistical differences were found when early and delayed H/M ratios were mutually compared. Combining the 2 radioisotopic procedures, a more reliable diagnosis was achieved in 39/43 cases properly classifying 13 PD, 10 VP, 7 PS, 5 LBD, and 4 AD. However, the diagnosis remained uncertain in four patients with normal ^123^I-ioflupane and reduced ^123^I-MIBG uptake. The results of the present study confirmed that in uncertain parkinsonian syndromes associated with vascular lesions in striatal nuclei, brain ^123^I-ioflupane SPECT alone did not prove able to discriminate between the different forms of disease. Only the association with ^123^I-MIBG cardiac scintigraphy, also with the early acquisition alone, allowed the most appropriate diagnosis in 90.7% of our cases. However, patients with normal ^123^I-ioflupane and reduced ^123^I-I-MIBG uptakes need a close clinical and instrumental follow-up as sympathetic damage could precede striatal disorders in the early stage of PD and LBD.

## 1 Introduction

The differential diagnosis of parkinsonian disorders represents a clinical dilemma especially in early phase, as clinical symptoms (slow movements, tremor, difficulty with walking and balance, stiffness, and rigidity) could be related to different conditions other than idiopathic Parkinson's disease (PD) such as progressive supranuclear palsy (PSP), multiple system atrophy (MSA), Lewy body dementia (LBD), cerebrovascular disorders, and iatrogenic drug effects.^[1,2]^ In particular, vascular parkinsonism (VP) due to vascular lesions mainly in the striatal nuclei, but also in white matter, is considered a distinct clinical form^[1,3]^ and the differential diagnosis between VP and PD is a crucial clinical point because of the difference in their progression, correct treatment strategy, potential supportive therapy, and prognosis.^[4,5]^

In the diagnostic criteria for suspected VP, there are included the clinical symptoms, the exclusion of Parkinson-plus syndromes, and other causes of secondary parkinsonism, while hypertensive cerebral vascular disease or previous history of transient ischemic attack or stroke, even if expected, are not considered as required parameters.^[6]^

Brain structural magnetic resonance imaging (MRI), evidencing vascular lesions, could be considered a supportive but not conclusive tool for confirming clinical diagnosis, as a specific pattern for VP has not yet been identified and some authors have underlined that ischemic brain lesions could be evidenced both in VP and PD cases^[3,7,8]^ as well as in Alzheimer dementia^[9]^ or in normal aged people with cardiovascular diseases or hypertension.^[10]^

Functional neuroimaging with ^123^I-ioflupane single photon emission computed tomography (SPECT), evaluating presynaptic striatal dopaminergic transporter with both qualitative and semiquantitative analyses,^[11]^ usually represents a useful tool both in PD initial diagnosis^[12,13]^ and in the differential diagnosis of uncertain parkinsonism.^[14,15]^ However, some authors evidenced that ^123^I-ioflupane SPECT was not able to differentiate PD from VP, being observed in some VP patients with vascular damage and unilateral disease an asymmetrical tracer striatal uptake similar to that evidenced in PD cases probably due to the limited tracer possibility of arriving to the binding sites.^[16]^ Therefore, PD diagnosis could be excluded when ^123^I-ioflupane SPECT was normal, but it remains unclear when a reduced tracer uptake occurs, thus leading to a diagnostic overlap.

^123^I-Metaiodobenzylguanidine (MIBG) cardiac scintigraphy represents a noninvasive method to evaluate postganglionic presynaptic cardiac sympathetic innervation.^[17,18]^ MIBG is an analog of the adrenergic-blocking agent guanethidine and has the same chemical structure that of norepinephrine; the chemical structure of the tracer allows its detection with an active mechanism by postganglionic presynaptic fibers, the storage in synaptic vesicles, and the release during nerve excitement with the same mechanism that of noradrenaline.

On the other hand, unlike noradrenaline, MIBG is not bound to cardiac receptors and is not degraded by the enzymatic systems catechol-*O*-methyl transferase and monoamine oxidase , but it is reabsorbed in the presynaptic side and accumulated in the vesicles nerve ending for a long time.

These features mean that ^123^I-MIBG represents the ideal radiotracer for the “in vivo” cardiac sympathetic system evaluation.

Moreover, the early ^123^I-MIBG cardiac uptake well correlates with denervation damage, whereas the evaluation of delayed phase, representing the tracer wash out, directly expresses the degree of sympatheticotonia.^[18]^

^123^I-MIBG cardiac scintigraphy was originally used to evaluate the presynaptic postganglionic endings of sympathetic system in a large number of cardiac diseases, such as congestive heart failure, ischemic heart disease, and cardiomyopathy.^[19–23]^ Afterwards, the procedure was applied in the diagnosis of PD^[24]^ and in the differential diagnosis of this disease from other neurodegenerative disorders, such as MSA, PSP, corticobasal degeneration,^[22,25]^ and VP.^[26,27]^

In this study we further evaluated the usefulness of ^123^I-ioflupane SPECT and ^123^I-MIBG cardiac scintigraphy combined use in clinical practice for differentiating clinically uncertain parkinsonian conditions associated with vascular cerebral lesions ascertained at MRI.

## 2 Material and methods

### 2.1 Patients

We evaluated retrospectively 43 consecutive patients, 19 males and 24 females aged 52 to 84 years (mean±SD value: 71.2 ± 9). All patients showed symptoms attributable to parkinsonian disorders associated with vascular cerebral lesions mainly in the striatal nuclei, but also in white matter ascertained at MRI; they were clinically classified as uncertain parkinsonism evidencing the presence of at least 1 of the following criteria: only 1 of the cardinal symptoms of PD, 2 clinical signs excluding bradykinesia, signs of mild intensity, atypical signs, poor response to l-dopa/dopamine agonist treatments, and lack of disease progression. The patients were investigated about neurological family history, about the presence of other related neurological diseases as well as about medical treatments such as antidepressants and corticosteroids, some of which can cause iatrogenic forms of parkinsonism. Exclusion criteria: metabolic disorders, such as renal and hepatic diseases, diabetes, and treatments affecting both ^123^I-ioflupane and ^123^I-MIBG uptakes. All patients had medical therapies for hypertension and hypercholesterolemia.

Table 1 illustrates the demographic, clinical, and treatment characteristics of the 43 patients.

**Table 1:**
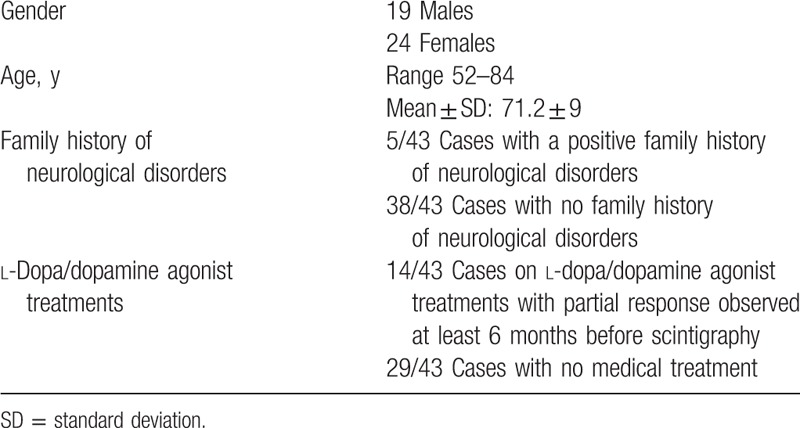
Demographic, clinical, and treatment characteristics of the whole group of 43 patients.

All patients underwent brain ^123^I-ioflupane SPECT and afterwards ^123^I-MIBG cardiac scintigraphy within 2 weeks from the former examination.

Before scintigraphic procedures, all patients were subdivided into 3 groups according to possible differential diagnostic hypotheses formulated on clinical and MRI data:

Group 1: with uncertain diagnosis between PD and VP (22 cases; 51.2%)
Group 2: with uncertain diagnosis between PD and other neurodegenerative parkinsonian syndromes (PS) (11 cases; 25.5%)
Group 3: with uncertain diagnosis between LBD and AD (10 cases; 23.3%)

### 2.2 Brain ^123^I-ioflupane SPECT

According to European Association Nuclear Medicine procedure guidelines,^[28]^ all patients were previously treated with 1000 mg of potassium perchlorate to minimize radiation exposure to the thyroid gland 30 minutes before the intravenous injection of 148 MBq of ^123^I-ioflupane (DaTSCAN, Amersham Health). Brain SPECT was performed 3 to 4 hours after the tracer injection by a dual-head gamma camera equipped with fan beam collimators (VG millennium; GE Healthcare). Acquisition and processing protocols had been previously prepared using a phantom for striatal imaging. The gamma camera was calibrated using the 159 keV photo peak ± 10% energy window. SPECT acquisition standardized parameters: rotation 180° for each head; frame size 128×128; zoom factor 1; frame time 30 seconds; and angular step 3°. SPECT images were acquired with the patients in the supine position and the head fixed in a holder.

SPECT data were normalized, processed by the back projection filter method applying a Butterworth filter (cut off frequency 0.5; order 10), and finally, a report of images, evaluated considering transaxial, coronal, and sagittal orbitomeatal-oriented slices (slice thickness: 2.23 mm), was obtained.

The images were qualitatively classified as normal (with striatal homogeneous and symmetrical intense tracer uptake in both caudate and putamen nuclei) or pathological (with striatal symmetrical or asymmetrical uptake defects);

In a postprocessing phase, a semiquantitative evaluation of the striatal ^123^I-ioflupane uptake was also performed by a dedicated software program (NEUROTRANS 3D; Segami Corp.), which defines striatal dopaminergic activity as binding potentials (BP) applying attenuation and partial volume effect corrections using a deformable 3D model segmentation generated by the degrading Talairach atlas. The BP cut-off of 3.3 for caudate and putamen was determined in 20 sex- and age-matched normal controls (mean values: 4.9 ± 0.71 and 4.6 ± 0.67 for caudate and putamen, respectively).

### 2.3 Cardiac ^123^I-MIBG scintigraphy

The EANM Cardiovascular Committee and the European Council of Nuclear Cardiology^[19]^ protocol was used to standardize both the acquisition and processing procedures.

Twenty-four hours before tracer injection and imaging, all patients were warned to stop taking drugs that could potentially interfere with catecholamine uptake, such as antidepressants, antipsychotics, and calcium channel blockers.^[19]^

In accordance with safety administration procedures, ^123^I-MIBG (111 MBq) was slowly intravenously injected with patients at rest in the supine position, after a weekly blood pressure monitoring, an overnight fast, and a thyroid blockade with oral administration of potassium perchlorate (1000 mg).

Cardiac planar imaging, in anterior–posterior and anterior-left oblique views, was acquired in all cases, 15 minutes (early) and 240 minutes (delayed) after tracer i.v. injection, by a dual-head gamma camera (INFINIA; GE Healthcare) equipped with low-energy, high-resolution, parallel hole collimators; the acquisition parameters were: matrix 128 × 128, zoom 1.4, time 600 seconds; the photopeak energy was centered at 159 keV with a window of 10%; the patients were in the same supine position in both acquisition phases.

Early and delayed images were evaluated by qualitative method and were considered normal with homogeneous tracer cardiac uptake and pathological with irregular inhomogeneous one. Qualitative analysis was supported by the semiquantitative data obtained calculating the heart to mediastinum (H/M) ratio by dividing the mean count density of left ventricle to those of the upper mediastinum (excluding the thyroid gland) deduced by regions of interest (ROIs) manually drawn in the anterior view both in early and delayed phases. The H/M ratio cut-off was set applying 2 different artificial classifiers, random forest and classification tree, on a large number of clinically ascertained cases;^[29,30]^ both statistical algorithms defined a cut-off of 1.56 in early and delayed phases.

Both ^123^I-ioflupane SPECT and ^123^I-MIBG cardiac scintigraphy were interpreted separately by 3 nuclear medicine physicians (SN, AS, and GM) who were informed of the clinical reason pertinent to the scintigraphy, but were unaware of the results of any investigations. Scintigraphic data were classified as normal with physiologic tracer distribution or pathological with scans evidenced of abnormal decreased tracer uptake in striatal nuclei and heart, respectively. Disagreements were resolved by consensus.

All clinical and instrumental examinations were performed in a University Hospital setting as part of the clinical care of neurodegenerative disease patients. This retrospective study was performed in accordance with the regulations of the Institutional Review Board and in accordance with the Declaration of Helsinki. Routinely, written informed consent had been obtained by all patients whose data were treated in accordance with the local privacy rules and regulations.

### 2.4 Statistical analysis

Student's *t* test for independent variables was utilized to compare early and delayed H/M ratios in normal and pathological cases. Statistical significance was considered as *P* <.05

## 3 Results

### 3.1 ^123^I-Ioflupane SPECT

Brain ^123^I-ioflupane qualitative analysis showed normal bilateral caudate and putamen tracer uptake in 9/43 cases (20.9%), 5 with PD/VP, 1 with PD/PS, and 3 with LBD/AD uncertain differential diagnosis, while it was evidenced pathological, slight to severe irregular reduced tracer uptake, in 34/43 (79.1%), 17 with PD/VP, 10 with PD/PS, and 7 with LBD/AD uncertain differential diagnosis.

In all the 9 normal cases, BP values were above cut-off value in both caudate and putamen nuclei, as illustrated in Table 2.

**Table 2:**
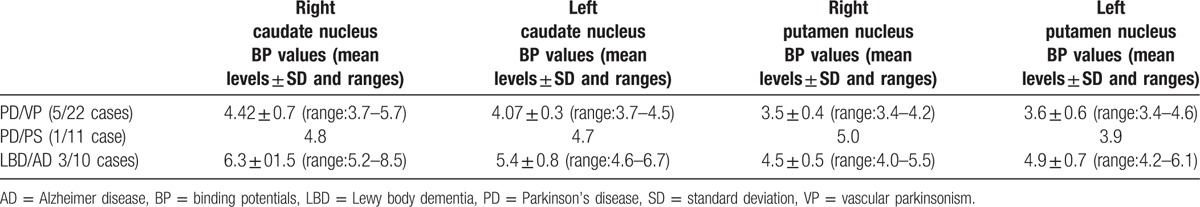
BP values (mean levels ± SD and ranges) obtained separately for each of caudate and putamen nuclei according to the diagnostic hypotheses in 9 normal ^123^I-ioflupane uptake cases.

In all the 34 pathological cases, BP values were under cut-off value at least in one of striatal nuclei when BP values obtained for each of caudate and putamen nuclei were considered subdividing these on the basis of different diagnostic hypotheses, as illustrated in Table 3.

**Table 3:**
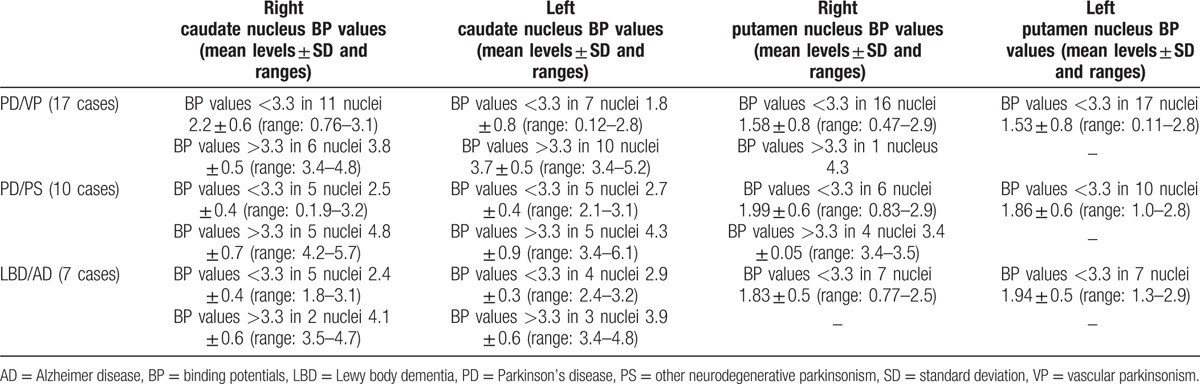
BP values (mean levels ± SD and ranges) obtained separately for each of caudate and putamen nuclei considered on the basis of the different diagnostic hypotheses in pathological ^123^I-ioflupane uptake cases.

In particular, considering separately each nucleus, it was observed that in right caudate BP values were low in 22/34 nuclei and they were included between 0 and 1 in 1 nucleus, between 1 and 2 in 4, and between 2 and 3.3 in remaining 17 nuclei; BP values were above cut-off value in 12/34 nuclei. In the left caudate, BP values were low in 16/34 nuclei and they were included between 0 and 1 in 1 nucleus, between 1 and 2 in 3, and between 2 and 3.3 in remaining 12 nuclei; BP values were above cut-off value in 18/34 nuclei.

In right putamen, BP values were low in 29/34 nuclei and these were included between 0 and 1 in 7 nuclei, between 1 and 2 in 15, and between 2 and 3.3 in remaining 10 nuclei; BP values were above cut-off value in 5/34 nuclei. In the left putamen, BP values were low in all 34 nuclei and these were included between 0 and 1 in 6 nuclei, between 1 and 2 in 15, and between 2 and 3.3 in remaining 13 nuclei.

### 3.2 ^123^I-MIBG cardiac scintigraphy

^123^I-MIBG cardiac scintigraphy qualitative evaluation showed, both in early and delayed phases, a homogenous tracer uptake in 21/43 patients (48.8%), 5 of whom had normal and 16 pathological ^123^I-ioflupane SPECT.

The H/M ratio values were >1.56, in both phases, in all 21 cases; no statistical difference was observed when early (1.71 ± 0.09) and delayed (1.76 ± 0.09) H/M ratio mean values were mutually compared, even when the patients were classified on the basis of ^123^I-ioflupane SPECT results (Table 4).

**Table 4:**
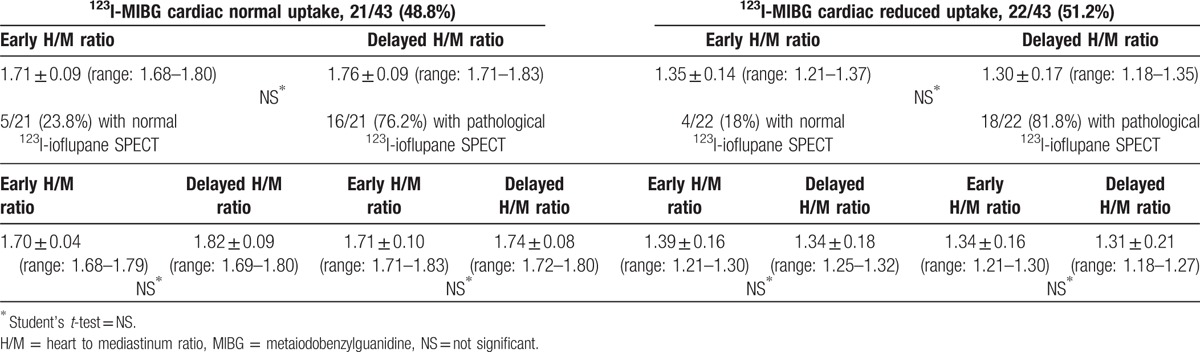
Qualitative analysis of ^123^I-MIBG cardiac scintigraphy results with early and delayed H/M ratio calculation in the 43 patients also subdivided on the basis of normal and pathological ^123^I-ioflupane striatal uptake.

^123^I-MIBG cardiac distribution showed slight to severe reduction in 22/43 cases (51.2%), 4 with normal and 18 with pathological ^123^I-ioflupane SPECT.

The H/M ratio values were <1.56, in both phases, in all 22 cases; no statistical difference was observed in the comparison between early (1.35 ± 0.14) and delayed (1.30 ± 0.17) H/M ratio mean values, even when patients were classified on the basis of ^123^I-ioflupane SPECT results (Table 4).

### 3.3 ^123^I-Ioflupane SPECT and ^123^I-MIBG cardiac scintigraphy combined use

The results obtained combining the 2 procedures have permitted to identify 4 different conditions:

A. Normal ^123^I-ioflupane SPECT and normal ^123^I-MIBG cardiac scintigraphy (5/43 cases; 11.6%)
B. Normal ^123^I-ioflupane SPECT and pathological ^123^I-MIBG cardiac scintigraphy (4/43 cases; 9.3%)
C. Pathological ^123^I-ioflupane SPECT and normal ^123^I-MIBG cardiac scintigraphy (16/43 cases; 37.2%)
D. Pathological ^123^I-ioflupane SPECT and pathological ^123^I-MIBG cardiac scintigraphy (18/43; 41.8%).

After ^123^I-ioflupane SPECT and ^123^I-MIBG cardiac scintigraphy results, a second clinical evaluation was performed to better define diagnosis.

Globally, when the data of the combined scintigraphic procedures were suggestive of normal or pathological conditions, the diagnosis was considered more reliable in 39/43 cases, whereas the diagnosis remained doubtful in those cases (4/43) with discordant scintigraphic results.

**Figure 1:**
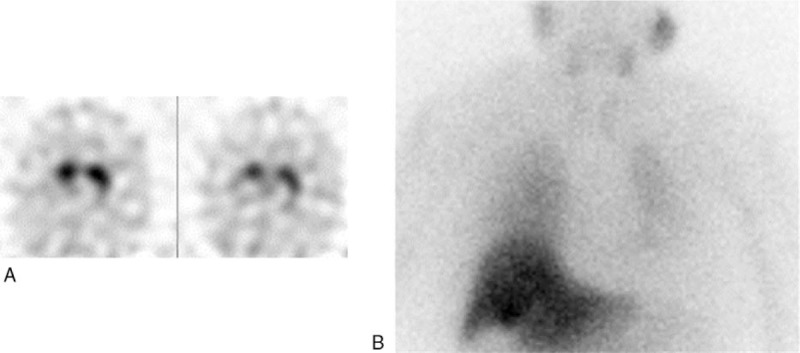
A 78-year-old female patient with uncertain parkinsonism and vascular lesions in subcortical areas, semioval centers, basal ganglia, and left temporal lobe at MRI. ^123^I-Ioflupane SPECT was pathological in both putamen nuclei (A) with also reduced BP values (1.7 and 1.8 in right and left putamen, respectively); ^123^I-MIBG cardiac scintigraphy (B) was pathological with reduced H/M values both in early (1.2) and delayed (1.10) phases. The patient was finally classified as PD. MRI= magnetic resonance imaging, SPECT = single photon emission computed tomography.

**Figure 2:**
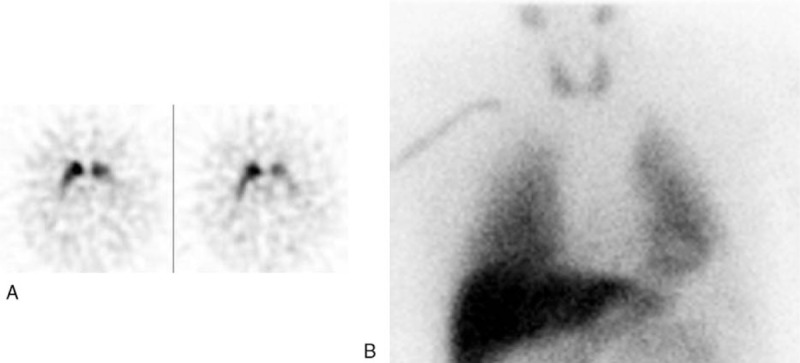
A 70-year-old male patient with uncertain parkinsonism, vascular lesions in basal ganglia at MRI, and partial response to dopamine agonist treatment. ^123^I-Ioflupane SPECT was pathological in left putamen nucleus (A) with also reduced BP value (1.9); ^123^I-MIBG cardiac scintigraphy was normal (B) with H/M values above cut-off both in early (1.8) and delayed (1.8) phases. The patient was finally classified as VP. H/M = heart to mediastinum ratio, MIBG = metaiodobenzylguanidine, SPECT = single photon emission computed tomography, VP = vascular parkinsonism.

**Figure 3:**
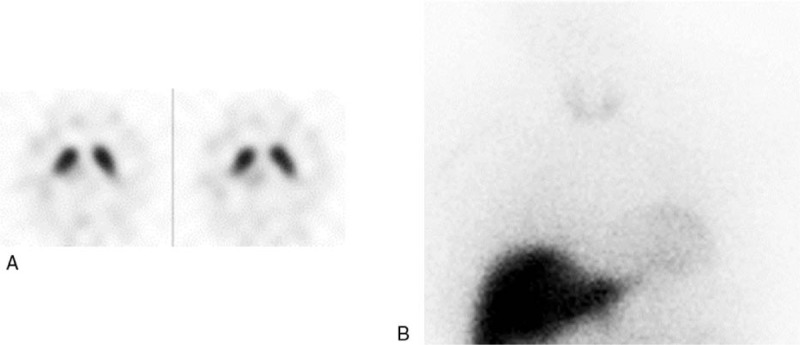
A 55-year-old male patient with uncertain parkinsonism, vascular lesions in left basal ganglia and midbrain at MRI, and partial response to dopamine agonist treatment. ^123^I-Ioflupane SPECT was normal (A) with also BP values above cut-off in caudate and putamen nuclei, bilaterally; ^123^I-MIBG cardiac scintigraphy was pathological (B) with reduced H/M values both in early (1.43) and delayed (1.38) phases. The patient was finally confirmed with uncertain parkinsonism and monitored in a close follow up. H/M = heart to mediastinum ratio, MRI= magnetic resonance imaging, MIBG = metaiodobenzylguanidine, SPECT = single photon emission computed tomography.

In particular, as shown in Table 5, among the 22 initially considered PD/VP uncertain cases, 19 (86.4%) were more properly classified: 9/22 cases with pathological ^123^I-ioflupane SPECT and pathological ^123^I-MIBG cardiac scintigraphy as PD (a case is illustrated in Fig. 1); and 10/22 cases with normal ^123^I-ioflupane SPECT and normal ^123^I-MIBG cardiac scintigraphy or pathological ^123^I-ioflupane SPECT and normal ^123^I-MIBG cardiac scintigraphy, as VP (a case is illustrated in Fig. 2). In the remaining 3/22 cases (13.6%), with normal ^123^I-ioflupane SPECT and pathological ^123^I-MIBG cardiac scintigraphy, the diagnosis was considered doubtful (a case is illustrated in Fig. 3).

**Table 5:**
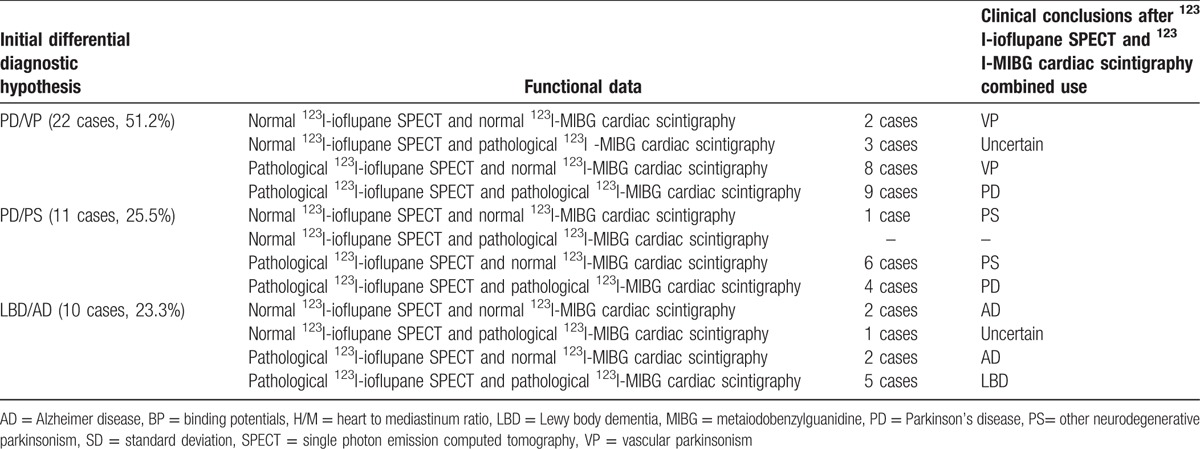
Initial differential diagnostic hypothesis and variation of clinical approach after ^123^I-ioflupane SPECT and ^123^I-MIBG cardiac scintigraphy combined use.

Of the 11 initially uncertain PD/PS patients, 4/11 with pathological ^123^I-ioflupane SPECT and pathological ^123^I-MIBG cardiac scintigraphy had finally a clinical diagnosis of PD. The remaining 7/11 with normal ^123^I-ioflupane SPECT and normal ^123^I-MIBG cardiac scintigraphy or pathological ^123^I-ioflupane SPECT and normal ^123^I-MIBG cardiac scintigraphy were classified as PS.

Among the 10 initially doubtful LBD/AD cases, 5/10 with pathological ^123^I-ioflupane SPECT and pathological ^123^I-MIBG cardiac scintigraphy were finally considered as LBD and 4/10 with normal ^123^I-ioflupane SPECT and normal ^123^I-MIBG cardiac scintigraphy or pathological ^123^I-ioflupane SPECT and normal ^123^I-MIBG cardiac scintigraphy as AD. The remaining 1 patient with normal ^123^I-ioflupane SPECT and pathological ^123^I-MIBG cardiac scintigraphy was confirmed as uncertain diagnosis.

All 4 patients classified as doubtful diagnosis are still monitored in a close follow-up.

## 4 Discussion

Parkinsonian syndromes often represent a clinical dilemma especially in the early stage and neuroimaging techniques are usually employed as complementary diagnostic tools in the initial differential diagnosis^[31]^ of disease.

^123^I-Ioflupane SPECT is a useful functional imaging in the diagnosis of PD and its high specificity suggests that the combined use of this procedure with the neurological examination in the initial evaluation of the disease could reduce the overdiagnosis of PD, thus limiting the use of inappropriate medical treatments.^[32]^ However, controversial data have been reported applying ^123^I-ioflupane SPECT in uncertain parkinsonian syndromes when multiple vascular lesions occur, especially in the few studies focalized on the differential diagnosis between PD and VP, the 2 most frequent clinical forms that need to be distinguished. In comparative studies between VP and PD patients, some authors^[33]^ pointed that ^123^I-ioflupane uptake may be useful in the differential diagnosis between PD and VP, observing significant statistical differences in striatal tracer binding in these 2 conditions. However, the same authors observed an overlap of the whole striatal binding in some PD and VP patients, thus reducing ^123^I-ioflupane SPECT accuracy. In addition, other authors^[34,35]^ have suggested caution in the use of ^123^I-ioflupane SPECT in the differential diagnosis between VP and PD, not being clearly yet determined, in their opinion, the patterns of tracer uptake for each disease. Furthermore, although ^123^I-ioflupane SPECT may represent a potential reliable tool also in the differential diagnosis between PD and PS and between AD and LBD, there are no data demonstrating its use in these disorders when vascular damage occurs.

The data of the present study, in respect of the previous mentioned studies that only included ascertained PD and VP cases, came from different groups of patients with uncertain parkinsonian syndromes and with vascular lesions mainly in the striatal nuclei, but also in white matter at MRI. Moreover, cases with differential diagnosis between PD and VP (PD/VP) were also included as well as patients with differential diagnosis between PD and PS (PD/PS) and between LBD and AD (LBD/AD). The results evidenced that ^123^I-ioflupane SPECT alone provided additional information, but not useful to effectively support the clinical differential diagnosis when cerebrovascular lesions occurred at MRI, as approximately 20% of our cases had normal scintigraphic data, whereas the remaining 80% showed pathological results, but not useful to clarify the differential diagnosis between the different forms of disease. These data emphasized the overlap of ^123^I-ioflupane uptake in the different clinical forms not only in PD/VP cases, in accordance with the results of other authors,^[33,34]^ but also in a wider number of other clinical forms, as reported in the present study probably in relation to the inability of the tracer to reach the presynaptic receptor level due to vascular disruption.

It is well known that ^123^I-MIBG cardiac scintigraphy, expressing the cardiac sympathetic postganglionic function, could be used both in the initial diagnosis of PD^[24]^ and in the differential diagnosis between PD and other neurodegenerative parkinsonism^[22,36]^ as well as in distinguishing LBD from AD.^[37]^ However, only a few studies,^[38–43]^ with different approaches, have evaluated the clinical utility of the combined use of the 2 procedures in the differential diagnosis of uncertain parkinsonian disorders; only one of these studies^[43]^ have reported the complementary role of ^123^I-MIBG cardiac scintigraphy in the patients with vascular lesions at MRI and, in particular, considering the differential diagnosis between VP and PD, as brain vascular lesions often can be responsible of uncertain results of ^123^I-ioflupane SPECT.

A contemporary damage in dopaminergic nigrostriatal system and in sympathetic cardiac neurons in PD was hypothesized^[38]^ obtaining a significant positive correlation between reduced ^123^I-ioflupane SPECT uptake and cardiac decreased uptake of ^123^I-MIBG in early PD cases with still normal MRI data. These results seem to suggest that the combined use of both ^123^I-ioflupane SPECT and ^123^I-MIBG cardiac scintigraphy in early diagnosis of PD may be clinically useful, especially when only slight symptoms occur; the clinical “in vivo” diagnosis is difficult to achieve in this conditions. An overlap between patients with PD and other parkinsonian syndromes, such as MSA, can also appear, as postmortem studies have ascertained.^[44,45]^

Some authors^[39]^ evaluated a multidimensional statistical approach of both ^123^I-ioflupane SPECT and ^123^I-MIBG cardiac scintigraphy; they also studied ^123^I-labeled dopamine D2 receptor ligands (IBZM) in the differential diagnosis of both PD and other atypical parkinsonism. The results of this study confirmed that there is a low diagnostic accuracy when the single procedures were used alone, evidencing an overlap between PD and PS. The same authors observed that the best diagnostic accuracy in distinguishing PD from PS was achieved combining the 3 procedures. However, in order to minimize cost and radiation exposure, the combined use of ^123^I-IBZM and ^123^I-MIBG scintigraphy should be suggested as the better choice as these 2 procedures evidence the striatal D2 receptor expression and the cardiac MIBG accumulation, respectively; the employment of ^123^I-ioflupane SPECT could, however, additionally be associated in borderline or contradictory results.

Other authors^[40–42]^ evaluated the role of the combined use of ^123^I-ioflupane SPECT and ^123^I-MIBG cardiac scintigraphy in patients with mixed tremors or in those cases in whom is necessary to differentiate LBD from other dementias. They suggested that the higher sensitivity of ^123^I-ioflupane SPECT and the higher specificity of ^123^I-MIBG cardiac scintigraphy could lead, when combined, to more accurate differential diagnosis, thus permitting the most appropriate treatments.

Our data, thought including a more complex series of patients in respect of the other mentioned casuistries, seem to confirm the results obtained by other authors^[43]^ in the only study that consider the combined use of the 2 procedures. In particular, in respect of the initial clinical evaluation and of the evaluation performed after ^123^I-ioflupane SPECT, the clinical diagnosis based on the results of the combined use of the 2 scintigraphic procedures was more correct in 90.7% of our cases. This result seems to confirm that ^123^I-ioflupane SPECT and ^123^I-MIBG cardiac scintigraphy combined use could represent a more valuable tool in the diagnosis of uncertain parkinsonian syndromes, also considering the easy availability of the radiopharmaceuticals and the rather limited radioprotection problems.

Considering the patients with normal ^123^I-ioflupane SPECT and normal ^123^I-MIBG cardiac scintigraphy as well as those with pathological ^123^I-ioflupane SPECT and normal ^123^I-MIBG cardiac scintigraphy, a final diagnosis was achieved as VP in 10 cases, PS in 7 cases, and AD in 4 cases. Moreover, pathological ^123^I-ioflupane SPECT and pathological ^123^I-MIBG cardiac scintigraphy enforced the diagnosis of PD in 13 patients and LBD in 5 cases.

Therefore, the combined use of the 2 radioisotopic procedures, supporting clinical diagnosis, have contributed to modify the treatment in those cases already treated, but with poor results, at the moment of scintigraphic exams and, at the same time, to guide to a more specific therapy those cases not yet treated.

In 4 cases (3 PD/VP and 1 LBD/AD), the 2 scintigraphic methods were inconclusive as ^123^I-MIBG cardiac scintigraphy was pathological despite normal ioflupane SPECT. These controversial results were also reported by other authors^[46,47]^ who hypothesized an early PD stage with no evident motor signs and with a striatal damage not yet manifest, but with cardiac sympathetic disorder, thus speculating an earliest involvement of autonomic system. We think that a similar mechanism could also be possible in the 3 our PD/VP cases and in the 1 LBD/AD case, having been excluded a priori all interfering factors in the 2 tracer uptakes. However, only a close follow up of the patients could confirm this hypothesis, and they are still monitored and are waiting of treatment. However, as often it happens, only autopsy could be determinant for final diagnosis.

Moreover, with regard to semiquantitative analysis of ^123^I-MIBG, our results have not evidenced statistically significant differences in the comparison between early and delayed H/M ratio mean values, regardless of patients classified on the basis of ^123^I-ioflupane SPECT results. For clinical neurological purposes, these data seem to suggest that the early phase alone could contribute to the diagnosis permitting also an easier patient management, according to the study of other authors^[48]^ who, evaluating a large series of LBD patients, achieved an accurate differential diagnosis between LBD and non-LBD cases only applying an early H/M ratio.

## 5 Conclusions

In conclusion, our results proved that in uncertain parkinsonian syndromes with vascular lesions in striatal nuclei and in white matter ascertained at MRI, ^123^I-ioflupane SPECT alone is not able to clearly differentiate PD from VP and from other parkinsonism as well as LBD from AD often showing an overlap in tracer uptake. Only the combined use of ^123^I-ioflupane SPECT and ^123^I-MIBG cardiac scintigraphy, also with the early acquisition of this latter alone, seems to give a more appropriate diagnosis in association with neurological examination, as in our cases, the results of these associated neuroimaging procedures gave useful information in more than 90% of cases. Moreover, in the presence of doubtful results with pathological ^123^I-MIBG uptake and normal ^123^I-ioflupane uptake, the patients must be monitored with a close follow up as sympathetic damage could precede striatal disorders in an early stage of Parkinson's or LBD diseases.

Thus, a wider employment of these 2 combined procedures is suggested when striatal vascular damage is associated with uncertain Parkinsonian syndromes.
